# Evaluating an intervention to promote access to mental healthcare for low language proficient migrants and refugees across Europe (MentalHealth4All): study protocol for a pretest–post-test cross-national survey study

**DOI:** 10.1136/bmjopen-2024-095868

**Published:** 2025-07-07

**Authors:** Liza G G van Lent, Sona Hodakova, Saskia Hanft-Robert, Mike Mösko, Chiara Rao, Koen Kerremans, Antoon Cox, Raquel Lázaro Gutiérrez, Özlem Temizöz, Dalia Mankauskienė, Łucja Biel, Emilio Di Maria, Barbara Schouten, MentalHealth4All consortium, Julia van Weert

**Affiliations:** 1 Communication Science, University of Amsterdam, Amsterdam, The Netherlands; 2 Translation Studies, Constantine the Philosopher University, Nitra, Slovakia; 3 Department for Medical Psychology, University Medical Center Hamburg-Eppendorf, Hamburg, Germany; 4 Applied Human Sciences, Magdeburg-Stendal University of Applied Sciences, Stendal, Germany; 5 Department of Linguistics & Literary Studies, Brussels Centre for Language Studies, Vrije Universiteit Brussel, Brussels, Belgium; 6 Translation Studies Research Unit, KU Leuven, Leuven, Belgium; 7 Department of Modern Philology, FITISPos-UAH Group, University of Alcala, Alcala de Henares, Spain; 8 Centre for Translation Studies, School of Languages and Literature, University of Surrey, Guildford, UK; 9 Institute for Literary, Cultural and Translation Studies, Faculty of Philology, Vilnius University, Vilnius, Lithuania; 10 Institute of Applied Linguistics, University of Warsaw, Warszawa, Poland; 11 University of Genoa, Genova, Italy; 12 University Unit of Medical Genetics, Galliera Hospital Trust, Genova, Italy; 13 Centre for Urban Mental Health, University of Amsterdam, Amsterdam, The Netherlands

**Keywords:** MENTAL HEALTH, Information technology, eHealth, Health Equity, Health Services Accessibility, PUBLIC HEALTH

## Abstract

**Abstract:**

**Background:**

Migrants and refugees with low language proficiency (LLP) in the dominant language of their host country have a higher risk of suffering from certain mental health disorders compared with non-migrant populations. They are also more likely to experience a lack of access to mental healthcare due to language-related and culture-related barriers. As part of the MentalHealth4All project, a digital multilingual communication and information platform was developed to promote access to mental healthcare for LLP migrants and refugees across Europe. This paper describes the study protocol for evaluating the platform in practice, among both health and/or social care providers (HSCPs) and LLP migrants and refugees.

**Methods and analysis:**

We will conduct a pretest–post-test cross-national survey study to evaluate the platform’s effect evaluation (primary objective) and process evaluation (secondary objective). The primary outcomes (measured at T0, T2 and T3) are four dimensions of access to mental healthcare services: availability, approachability, acceptability and appropriateness of mental healthcare. Secondary outcomes (measured at T2) are: actual usage of the platform (ie, tracking data), perceived ease of use, usefulness of content, comprehensibility of information, attractiveness of content and emotional support. Participants will be recruited from nine European countries: Belgium, Germany, Italy, Lithuania, the Netherlands, Poland, Slovakia, Spain and the UK. Using convenience sampling through professional networks/organisations and key figures, we aim to include at least 52 HSCPs (ie, 6–10 per country) and 260 LLP migrants (ie, 30–35 per country). After completing a pretest questionnaire (T0), participants will be requested to use the platform, and HSCPs will participate in an additional personalised training (T1). Next, participants will fill out a post-test questionnaire (T2) and will be requested to participate in a second post-test questionnaire (T3, about 6–8 weeks after T2) to answer additional questions on their experiences through a brief phone interview (T3 is optional for migrants/refugees).

**Ethics and dissemination:**

For all nine countries, the ethical review board of the participating university (hospital) has assessed and approved the protocol. If successful, the MentalHealth4All platform will be made publicly available to help improve access to mental healthcare services, as well as HSCPs’ cultural competencies in delivering such services, for any LLP migrants and refugees across Europe (and beyond). Findings will also be disseminated through peer-reviewed journals and conferences.

**Registration details:**

The ‘MHealth4All project’ was prospectively registered on Open Science Framework, DOI: 10.17605/OSF.IO/U4XSM.

**STRENGTHS AND LIMITATIONS OF THIS STUDY**

- The broad geographical and linguistic coverage of the MentalHealth4All consortium ensures recruitment of diverse groups of low language proficiency (LLP) migrants and refugees and health and/or social care providers (HSCPs).
- The complementary skills, expertise and experiences of the consortium partners in working and collaborating with LLP migrants and refugees enable us to draw on existing collaborations for data collection.
- Although this study’s main challenge concerns the recruitment/drop-out of LLP migrants and refugees, previous studies in the project have proven the feasibility of including these populations in our research.
- To ensure implementation in practice, HSCPs follow a webinar and personalised training session in which they learn how to (overcome barriers to) integrate the platform in their daily practice.
- The appropriateness of performing sensitivity analyses with only migrants who experience mental health issues and of exploring differences across countries depends on the final sample.

## Introduction

 Mental health is a vital part of one’s well-being.^1^,^3^ Yet, about one billion people worldwide suffer from (diagnosable) mental health disorders.^1 4^ Migrants and refugees are at increased risk of suffering from specific disorders such as post-traumatic stress, depression and psychosis-related disorder, with higher prevalence rates compared with those of non-migrant populations.^5^,^9^ Yet, migrants’ and refugees’ access to mental healthcare services is often severely hindered,^10^,^16^ because of a combination of cultural barriers such as mental health stigma and taboo, and structural barriers such as low language proficiency (LLP) in the dominant language(s) of their country of residence, a lack of information about the healthcare system in one’s mother tongue and (perceived) discrimination.^10 11 17 18^ Language barriers between clients and their health and/or social care providers (HSCPs) who do not share a common language form one of the main factors contributing to persisting health inequalities in access to (mental) healthcare.^19^ Notwithstanding international human rights standards to reduce health inequalities,^20^ the European Commission has called for more attention to integration measures for refugees and migrants in Europe, in particular concerning access to (mental) healthcare services.^21^

With the number of migrants and refugees at unprecedented and still increasing levels worldwide,^22 23^ responding to the call made by the European Commission and promoting their access to mental healthcare is of crucial importance. To achieve this, digital or technology-based interventions could provide a necessary partial solution.^11 24 25^ Digital interventions have several advantages over non-digital interventions, among which wider access to expert care and the possibility to use various modalities suited to different literacy levels.^26^ Previous studies have shown that—particularly cocreated^27^—(digital) interventions have successfully enhanced healthcare access and health outcomes in marginalised populations.^28^,^31^ Building on these successes, our platform is designed to address the unique challenges faced by LLP refugees and migrants who suffer from mental health problems. Specifically in the context of promoting access to mental healthcare for LLP migrants and refugees, digital interventions could build on recommendations from previous studies by offering culturally sensitive multilingual materials,^32^ including all stakeholders in a co-creation process^33^ and also offering support to providers who refer to and/or offer mental health services.^34^

Despite their great potential, evidence-based digital interventions to promote access to mental healthcare services for these target groups across Europe are still very scarce and often not properly evaluated in terms of their effectiveness.^35^ The MentalHealth4All project aims to fill these gaps by designing and evaluating a culture-sensitive multilingual digital platform to promote this access, which is defined as ‘the *opportunity* to use health services, reflecting an understanding that there is a set of circumstances that allows for the use of appropriate health services’.^36^ This evaluation study will primarily focus on four dimensions of access, namely: availability (ie, the presence and capacity of facilities), approachability (ie, access to information on (patient) rights, services available and costs of services), acceptability (ie, cultural competence by providers) and appropriateness (ie, how well the services provided match the needs of the refugee/migrant populations).^37 38^ Building on insights from previous parts of the project^14 39 40^ and following the Spiral Technology Action Research (STAR) Model,^41^ an evidence-based sustainable digital information and communication platform was cocreated with LLP migrants and refugees in order to promote their access to mental healthcare on these four dimensions.

This study protocol describes the final part of the MentalHealth4All project, which aims to prospectively evaluate the intervention in practice across nine European countries. The primary objective of this study is the effect evaluation, that is, to evaluate the effects of the digital platform on the main dimensions of perceived access to mental healthcare services.^37 38^ The secondary objective of this study is the process evaluation, that is, to evaluate the digital platform with regard to the process of implementing the platform in healthcare practice. Corresponding with these objectives, we will answer the following research questions:

*RQ1*. How does the digital platform affect participants’ (ie, migrants/refugees and HSCPs) perceptions of access to mental healthcare in terms of: (1) availability, (2) approachability, (3) acceptability and (4) appropriateness?

*RQ2*. How do participants (ie, migrants/refugees and HSCPs) use the digital platform in terms of the pages/videos they access and average duration of engagement?

*RQ3*. How do participants (ie, migrants/refugees and HSCPs) evaluate the digital platform in terms of: (1) ease of use, (2) usefulness of the content, (3) comprehensibility of the information, (4) attractiveness of content and (5) emotional support?

## Methods and analysis

### Study design

To answer the research questions, a pretest–post-test cross-national study will be conducted to evaluate the MentalHealth4All digital platform in nine European countries (ie, Belgium, Germany, Italy, Lithuania, the Netherlands, Poland, Slovakia, Spain or the UK). These countries were selected as consortium partners to ensure broad geopolitical representation across northern, eastern, southern and western Europe. Due to the nature of the study design and intervention, either randomising or blinding participants is not possible. We will apply an intention-to-treat approach,^42 43^ that is, including participants regardless of how long they have used the digital platform. This approach aligns well with actual practice where, for instance, some people may search for specific information and therefore briefly use a specific part of the platform, while others may go through the entire platform to explore what possibilities there are. For each country, the ethical review board of the participating university/hospital has assessed and approved the protocol.

### Study population

For this study, we aim to include two main groups of participants: (1) LLP migrants and refugees and (2) HSCPs who have experience in providing (mental) healthcare for LLP migrants and refugees.

#### Inclusion criteria

The following inclusion criteria will be adhered to for migrants and refugees:

- Currently living in one of the consortium’s participating countries (ie, Belgium, Germany, Italy, Lithuania, the Netherlands, Poland, Slovakia, Spain or the UK).
- Refugee/migrant background originating outside the country of residence.
- ≥18 years old.
- Limited proficiency in the dominant language(s) of the country of residence (and therefore experiencing language barriers) as verified with a self-reported scale in the questionnaire.
- Sufficiently proficient to understand (at least) 1 of the 15 languages of the multilingual digital platform (ie, Arabic, Chinese, Dutch, English, French, German, Italian, Lithuanian, Persian, Polish, Russian, Slovak, Spanish, Turkish and Ukrainian).

For inclusion of the HSCPs, the following inclusion criteria will be used:

- Experience in delivering (mental) healthcare to migrants and refugees (ie, at least one consultation with an LLP refugee/migrant client per month on average in the past 6 months).
- Currently working in one of the participating European countries (ie, Belgium, Germany, Italy, Lithuania, the Netherlands, Poland, Slovakia, Spain or the UK).
- Sufficiently proficient to understand (at least) 1 of the 15 languages of the multilingual digital platform.

#### Exclusion criteria

The exclusion criterion for all participants is:

- No access to the internet (in order to use the digital platform) and impossible to arrange this (eg, at one’s medical/community centre).

### Intervention

The intervention around which this evaluation study revolves is a digital platform aimed at HSCPs and LLP migrants and refugees (as well as their (informal) caregivers). The platform was developed following the STAR model^41^ for eHealth interventions, which provided a rigorous participatory engagement model to optimise the involvement of these end users. The model consists of five phases (‘listen’, ‘plan’, ‘do’, ‘study’ and ‘act’). First (‘listen’), a mixed-method approach was applied to map resources as well as the most severe barriers, salient needs and recommended communication strategies for both HSCPs and LLP migrants and refugees.^14 39 40^ Second (‘plan’), audiovisual communication strategies in the form of animated videos were developed in Vyond for LLP migrants and refugees^44^ and for HSCPs (*publication in progress*). Third (‘do’), building on these insights from previous parts of the project, the platform was developed. The current study focuses on the fourth and fifth phases (‘study’ and ‘act’) of the STAR model^41^ in which the intervention will be implemented and evaluated in practice.

The platform (see: www.mentalhealth4all.eu (pending positive results, the platform will only be available for participants with a personal code for the duration of the current study)) consists of different informative elements, related to the different dimensions of access, including:

- Availability: a map of Europe that shows the presence of several organisations that provide (culturally sensitive) mental healthcare.
- Approachability: one set of eight videos aimed at LLP migrants and refugees with information on, for example, available services, mental health conditions and the healthcare system.^44^
- Acceptability: one set of 10 videos aimed at HSCPs, for example, about working with interpreters, communication strategies and recognising/addressing mental health issues in migrants and refugees (*publication in progress*).
- Appropriateness: a resource repository/information portal where users can select/search for the information they need from an exhaustive set of multilingual materials such as documents or videos about mental health or language services.^40^

The platform can be used on different devices, including computers/laptops, tablets and smartphones. For the current evaluation study, the platform will be available in fifteen languages: Dutch, English, French, German, Italian, Lithuanian, Polish, Slovak, Spanish (ie, the dominant languages of the participating countries), Arabic, Chinese, Persian, Russian, Turkish and Ukrainian (ie, languages representing large migrant language groups in Europe).

For the HSCPs, we have developed an accompanying, standardised training to teach them how to work with the platform and how to integrate it into their clinical practice. The training consists of two parts:

1. A general online webinar for HSCPs on how they can use the platform in practice—which is uploaded on the platform and available in the dominant languages of the nine participating countries.
2. A personalised, structured training session (either individual or in a group, and online or in-person) in which more detailed discussions will be held with a trainer on how to implement the (content of the) platform in the HSCPs’ daily practice, for which a standard training outline with a model presentation was developed to promote consistency across trainers/countries.

### Patient and public involvement

LLP migrants and their representatives have been involved in the entire MentalHealth4All project, including the original project proposal and (the preparations for) the current study. For example, the Foundation for the Health of Immigrants in the Netherlands has been involved as a consortium partner since the start of the project. Moreover, different platform components were cocreated with and/or pilot tested by LLP migrants as well as providers. Migrant representatives will also be involved in spreading the word about the project/study among other LLP migrants and refugees.

### Procedures

This study will be carried out in nine countries represented in the MentalHealth4All consortium: Belgium, Germany, Italy, Lithuania, the Netherlands, Poland, Slovakia, Spain and the UK. In all nine countries, we will use convenience sampling by approaching HSCPs through our existing networks and by reaching out to relevant professional organisations (eg, psychologists, social workers). Each HSCP will be asked to approach migrants and refugees who are, for example, on a waiting list or have only recently started treatment to participate in the study. Additionally, migrants and refugees will be recruited via key figures in communities that cover the target audience of this study, for example, social workers in neighbourhood facilities, cultural/religious leaders, general practitioners/family doctors and other prominent figures in social contexts. Besides, messages across refugee/migrant social media pages, migrant/refugee/professional organisations and the project’s social media channels will call for participants who meet the relevant criteria. Migrants and refugees who are interested can voluntarily reach out to the research team. We will also ask participants (migrants/refugees and HSCPs) if they may know others who would want to participate in this study (ie, ‘snowballing’). Before participation, all migrants/refugees and HSCPs will receive an information factsheet about the study and an informed consent form. We will use easy-to-understand information materials in multiple languages, that is, in the participants’ mother tongue or a possible second language. Multilingual research assistants will be available to help (verbally) discuss the information in participants’ mother tongue and answer possible questions.

**Figure 1:**
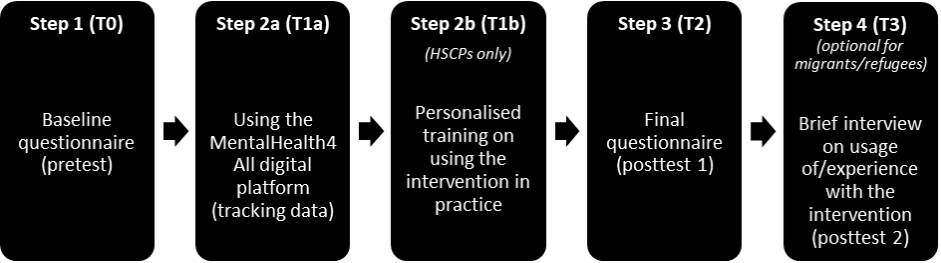
Participation process. HSCPs, health and/or social care providers.

After agreeing to participate, participants will receive the link to the questionnaires (see online supplemental appendix 1) and a personalised log-in code (for both the questionnaires and the platform). Participants will then be asked to go through three different steps, as illustrated in figure 1. First, participants will be asked to fill out the pretest questionnaire to establish a baseline, which starts by asking informed consent (T0). Second, they will be asked to use the multilingual digital platform/intervention (T1; ie, for HSCP participants, this also includes participation in personalised training). Third, participants will be asked to fill out the post-test questionnaire to evaluate the platform’s effects on the four dimensions of access to mental healthcare immediately after having used the platform (T2). Fourth, after about 6–8 weeks after T1, HSCPs will participate in a brief phone or online interview to answer some final questions on their usage of/experiences with the digital platform and its effects on access to mental healthcare (T3). The digital platform and the questionnaires will be available online in multiple languages in order to match participants’ mother tongue or a possible second language. If a participant prefers, the surveys could be administered on paper or verbally, for instance, by a (multilingual) research assistant or with the possible help of an interpreter.

### Measurements

All measurements can be found in online supplemental appendix 1. All consortium partners agreed on the content, and measurements were translated into the languages of the platform, either by human translators following current ISO standards or by using Microsoft Translator for automatic translations that were carefully checked/corrected by native speakers. The questionnaires and platform all start by filling out a personalised code in order to link the surveys to platform usage. Only the principal and executive investigators at the university in a participant’s country of residence will be able to link a personal code to a participant’s identity.

#### Primary outcome measurements

In line with RQ1, the main study parameter is access to mental healthcare services, measured by means of four dimensions, that is, availability, approachability, acceptability and appropriateness.^37 38^ These four dimensions are the main endpoints for this study. Based on other existing questionnaires, we selected and/or developed our own questions to assess these dimensions. In particular, we used existing scales for mental health literacy,^45^,^47^ and the Canadian Community Health Survey on accessibility, acceptability and availability^48^ as inspiration for these endpoints. Additionally, we supplemented the dimensions with statements that were considered of particular relevance in the current context (ie, migrants/refugees and mental health) based on the extensive expertise of the project team. These primary outcome measurements will be assessed at T0 (pre test), T2 (first post test) and T3 (second post test).

- *Availability of care*: the presence and capacity of facilities will be assessed with five items on a five-point Likert scale (1=strongly disagree, 5=strongly agree).
- *Approachability of care*: this dimension entails access to information on (patient) rights, services available and costs of services and will be assessed with seven items on a five-point Likert scale (1=strongly disagree, 5=strongly agree).
- *Acceptability of care*: this dimension assesses providers’ cultural competence and will be assessed with four items on a five-point Likert scale (1=strongly disagree, 5=strongly agree).
- *Appropriateness of care*: this dimension indicates how well the services provided match the needs of the refugee/migrant populations and will be assessed with six items on a five-point Likert scale (1=strongly disagree, 5=strongly agree).

#### Secondary outcome measurements

The secondary study parameters are related to the process evaluation of the platform (RQ2 and RQ3), in particular, the animated videos and the resource repository/information portal and the HSCP training, which will be measured at T2 (first post test) and at T1a (by tracking the actual usage of the digital platform). The secondary endpoints for this study are:

- *Actual usage of the digital platform*: we will monitor participants’ actual usage of the platform by means of a built-in tracker. In particular, we will collect data on the time spent in total and on each separate page, the number of visits, the number of clicks and the videos that were watched.^49^ Each participant will receive a unique personal code to log into the digital platform. This will enable us to connect website usage to other outcomes from the surveys.
- *Ease of use*: inspired by the Extended Unified Theory of Acceptance and Use of Technology (UTAUT2),^50 51^ we will use the three items of the ‘effort expectancy’ subscale to measure the ease of use of the digital platform. For ease of administration (especially when the surveys have to be verbally administered by a research assistant) and consistency, we will assess all items on a five-point Likert scale (1=strongly disagree, 5=strongly agree), rather than the original seven-point Likert scale.
- *Usefulness of the content*: inspired by the UTAUT2,^50 51^ we will use two items from the ‘performance expectancy’ subscale to measure the usefulness of the digital platform. For ease of administration (especially when the surveys have to be verbally administered by a research assistant) and consistency, we will assess all items on a five-point Likert scale (1=strongly disagree, 5=strongly agree), rather than the original seven-point Likert scale.
- *Comprehensibility of the information*: we will use the three items from the subscale ‘satisfaction with comprehensibility’ of the Website Satisfaction Scale.^52^,^54^ For ease of administration (especially when the surveys have to be verbally administered by a research assistant) and consistency, we will assess all items on a five-point Likert scale (1=strongly disagree, 5=strongly agree), rather than the original seven-point Likert scale.
- *Attractiveness of the content*: we will use the five items from the subscale ‘satisfaction with attractiveness’ of the Website Satisfaction Scale.^52^,^54^ For ease of administration (especially when the surveys have to be verbally administered by a research assistant) and consistency, we will assess all items on a five-point Likert scale (1=strongly disagree, 5=strongly agree), rather than the original seven-point Likert scale.
- *Emotional support*: We will use the four items from the subscale ‘satisfaction with emotional support’ of the Website Satisfaction Scale.^52^,^54^ For ease of administration (especially when the surveys have to be verbally administered by a research assistant) and consistency, we will assess all items on a five-point Likert scale (1=strongly disagree, 5=strongly agree), rather than the original seven-point Likert scale.

#### Other (control) measurements

For all participants, the baseline/pretest questionnaire (T0) will include sociodemographic items that inquire about participants’ age, gender, education level, native country and country of residence. For the HSCPs, we will ask additional questions about their working and postgraduate training experiences, in particular with migrants and refugees. For migrants and refugees, we will ask about the main reason for leaving their native country and how long they have been living in their current country of residence. Furthermore, we will measure the following potentially confounding factors at T0 (pre test):

- *Mental health status* (only for LLP migrants/refugees): we will use the Mental Health Inventory-5 (MHI-5) to measure participants’ mental health status,^55^ which is part of the Short Form 36-Item Health Survey (SF-36).^56^ The MHI-5 consists of five items, which we will assess on a five-point scale (1=none of the time, 5=all of the time).
- *Health literacy*: the ability to perform the basic reading and numerical tasks required to function in the healthcare environment will be assessed using the set of brief screening questions (SBSQ).^57^ The SBSQ consists of three items on a five-point scale (1=never/not at all confident, 5=always/extremely confident).
- *eHealth literacy*: using the eHealth Literacy Scale (eHEALS),^58^ we will measure participants’ knowledge, comfort and perceived skills at finding, evaluating and applying electronic health information to health problems. The eHEALS consists of eight items on a five-point Likert scale (ie, 1=strongly disagree, 5=strongly agree).
- *Language proficiency in the main country of residence’s language*: we will ask participants to rate their proficiency in the main language of their country of residence using four items (ie, speaking, listening, reading and writing) on a five-point scale (ie, 1=not at all fluent, 5=completely fluent).

At T2 (first post test), we will measure the following parameters for all participants:

- *Technology acceptance*: in order to measure participants’ general acceptance of health websites, we will use two items from the ‘performance expectancy’ subscale, and the subscales ‘effort expectancy’ and ‘behavioural intention’ of the UTAUT2,^50 51^ totalling to eight items. For ease of administration (especially when the surveys have to be verbally administered by a research assistant) and consistency, we will assess all items on a five-point Likert scale (1=strongly disagree, 5=strongly agree), rather than the original seven-point Likert scale.
- *Language proficiency in the platform language*: we will ask participants to rate their proficiency in the language in which they used the multilingual digital platform using four items (ie, listening and reading) on a five-point scale (ie, 1=not at all fluent, 5=completely fluent).

The questionnaire at T2 (first post test) will also include some open-ended questions on the digital platform (ie, what participants liked and disliked, what they think could be added to the platform and other comments/thoughts on the platform). At T3 (second post test), additional open-ended questions will be asked on how HSPCs and migrants/refugees have used the intervention in practice.

### Sample size calculation

Based on an a priori power analysis using G*power V.3.1, with effect size set at f2 of 0.2, p value of <0.05 and power of 0.80 and number of groups and measurements at 2 (Analysis of Variance/ANOVA, repeated measures within subjects), a sample size of n=52 is required. Therefore, we will sample at least 52 HSCPs across all nine consortium countries. Additionally, we strive to include around five times as many LLP migrants and refugees (n=260) to ensure more diverse representation of migrants across different European countries and to ensure sufficient power for, for example, sensitivity analyses and including relevant covariates. In each country, we aim to include about 6–10 HSCPs and 30–35 migrants and refugees (to account for possible dropout).

### Statistical analysis

The latest version of IBM SPSS Statistics will be used to statistically analyse the data. The data analysis will be performed by researchers from the project lead of this work package, that is, the University of Amsterdam. For the analysis of the primary endpoints, that is, the four dimensions of access to mental healthcare, we will perform one-sided t-tests and repeated measure ANOVAs to evaluate differences in the measurements between the pre test (baseline survey) and post tests per group of participants (ie, migrants/refugees and HSCPs separately). Additionally, we will perform ANOVAs to check for possible covariates. If possible, we will perform sensitivity analyses with only the migrants/refugees who are currently experiencing self-reported mental health issues and explore differences between the participating countries. We will use a significance level of p≤0.05 for all analyses. Descriptive statistics will be provided for the secondary endpoints. Additionally, descriptive statistics and frequency distributions will also be generated for the participants’ sociodemographics.

## Ethics and dissemination

The ethical review boards of all participating universities have assessed and approved the protocol before the start of the study: University of Amsterdam, the Netherlands (FMG-7525, project lead), Vrije Universiteit Brussel (EC-2022-383), University Medical Center Hamburg-Eppendorf, Germany (LPEK-0740), University of Genova, Italy (2024/28), Vilnius University, Lithuania (indicated on inquiry that they did not require approval for this type of study), University of Warsaw, Poland (288/2024), the Constantine the Philosopher University, Slovakia (UKF-2022/842-2:191013), University of Alcalá, Spain (CEIP/2024/3/059) and University of Surrey, UK (FASS 23-24035 EGA).

The broad European geographical coverage of the MentalHealth4All consortium ensures wide dissemination of the project and recruitment of diverse groups of migrants and refugees. The results from the current evaluation study could thereby prove relevant for other migrant populations residing in one of the member states of the European Union and possibly beyond. We intend to disseminate the results of the current study by sharing the results with stakeholders and organisations who participated in the project. In line with the benefits of co-creation,^59 60^ the project has been discussed regularly with representatives of healthcare and migrant organisations who have all expressed their enthusiasm and willingness to cooperate with developing and implementing the platform in practice. Additionally, we plan to publish and present our findings in peer-reviewed journals and at (inter)national conferences. At last, we will organise an international symposium in Brussels related to this project in order to bring together academics, (mental) healthcare professionals, policymakers and members of civil society organisations.

## Discussion

This manuscript describes the study protocol for evaluating an innovative platform (with additional training for HSCPs) that provides information on (accessing) mental healthcare and language support in 15 languages. With this intervention, we aim to improve access to mental healthcare for migrants and refugees across nine European countries (which is currently often hindered^10^,^16^). By means of a pretest–post-test study, we will perform a large-scale process evaluation and effect evaluation of the intervention across nine European countries. Although this study’s main challenge may concern the recruitment and possible dropout of LLP migrants and refugees, the previous work packages in the MentalHealth4All project^14 39 40^ have already proven the feasibility of including these populations in our research. In particular, the findings of this pretest–post-test study will help to deepen our understanding of how to promote access to mental healthcare services for LLP migrants and refugees. If successful, this intervention could be used to improve (access to) mental healthcare services for any LLP migrants and refugees across Europe (and beyond).

## Supplementary material

10.1136/bmjopen-2024-095868 online supplemental appendix 1
