# Supplementary material for: Evaluating an intervention to promote access to mental healthcare for low language proficient migrants and refugees across Europe (MentalHealth4All): study protocol for a pretest–post-test cross-national survey study
Source: BMJ Open. 2025 Jul 7;15(7):e095868. doi: 10.1136/bmjopen-2024-095868 (PMC12258366; doi:10.1136/bmjopen-2024-095868)
Supplement: online supplemental appendix 1 [file bmjopen-15-7-s001.docx]

*In this Appendix, we describe the content of all questionnaires. Some points of attention:*

- *In order to enable the researchers to link the data of participants (anonymously), the questionnaire will be made available by using a personal log-in code, which participants (both TCN and HSCP) can also use to log onto the digital platform. This log-in code will also provide participants with the opportunity to pause the questionnaire and continue later.*
- *All questions are included in English, but will be translated into multiple languages in order to match the language of the host country (for HSCPs) and/or TCNs’ mother tongue.*
- *Some questions will only be asked to either HSCP or to TCN participants. If this is the case, this is indicated with the corresponding questions. When nothing is indicated, the question will be asked to all participants.*
- *In case (validated) scales are used, the name of these scales is mentioned in this document for the sake of completeness and clarity (i.e. in the grey rows). However, these scale names will not be shared with the participants – they will only see the questions/statements with the explanations.*
- *Depending on the preferences of the participants, the questionnaires can be administered online or verbally (i.e. with the help of a research assistant with whom the participant shares a language).*

# pretest questionnaire

## Start page

| Dear Sir/Madam,  Thank you for your interest in participating in the MHealth4All study project. This project is sponsored by the European Union and is conducted in nine European countries (namely: Belgium, Germany, Italy, Lithuania, the Netherlands, Poland, Slovakia, Spain, and the United Kingdom). The goal of the project is to evaluate the effects of a newly developed digital platform. In particular, we want to know if the platform can help improve access to mental healthcare for refugees and other migrants.  You have received this questionnaire because you are a [(mental) health and/or social care provider with (some) experience in caring for refugees or migrants]/[refugee or migrant’] in one of the participating European countries. With this first questionnaire, we would like to know more about *your* personal ideas and beliefs on mental healthcare in the country you are currently in. We would like to request you to fill out this questionnaire before using the digital platform (and before you participate in the additional training). The second questionnaire will focus on your experiences with the digital platform and its possible effects. There are no ‘right’ or ‘wrong’ answers. You would help us a lot by filling out this questionnaire using your first instinct or impressions. This questionnaire will take around 10-15 minutes to complete. People will not be able to trace your answers back to you or someone you care for.  We realize that the questionnaire may possibly evoke some emotions for you. If you feel upset or distressed, you do not have to continue with the questionnaire, or you can finish it another time. If you have filled out the questionnaire and feel the need to talk to someone, we advise you to contact a relative or friend, or your general practitioner/family caregiver.  Participation is voluntary. You are free to withdraw from the study at any given time. This will not disadvantage you in any way.  At the end of the questionnaire, there is the possibility to note additional thoughts or comments. If you have any questions or remarks about the study, please contact one of the researchers. Their contact details are below.  We greatly appreciate your participation and efforts.  Kind regards, on behalf of the MHealth4All study team,  [To be determined contact details per country]  More information about the MHealth4All project can be found on: <https://www.mhealth4all.eu/>. |
| --- |

## Start of the questionnaire

|  | **Statement** | **Please tick each box to confirm**  (N.B. un-ticked boxes will mean that you DO NOT agree to participate) |
| --- | --- | --- |
| 1 | I confirm that I have read and understood the participant information sheet for the MHealth4All study project. I have had the opportunity to consider the information and to ask questions which have been answered satisfactorily. I have had sufficient time to decide to participate in this study. |  |
| 2 | I understand that my participation is voluntary and that I am free to withdraw from the study while completing the questionnaire(s) without giving any reason. If I decide that I do not wish to be included in the study after I have submitted the questionnaire, I can contact the research team using the contact details at the end of the participant information sheet. |  |
| 3 | I understand that information I provide may be subject to review by responsible individuals from the University of Amsterdam and/or regulators for monitoring and audit purposes. |  |
| 4 | I understand that information I provide will be used in various anonymised outputs, including (scientific) publications and content for the website of the project. |  |
| 5 | I understand that my personal data, including this consent form, which link me to the research data, will be kept securely in accordance with data protection guidelines, and only be accessible to the immediate research team or responsible persons at the University. |  |
| 6 | I understand that any personal contact details collected about me, such as my e-mail address, will not be shared beyond the study team. |  |
| 7 | I agree to participate in this study. |  |

## Access to mental healthcare

| Below are a few statements related to accessing (mental) healthcare for refugees/migrants in the country where you live. All of these statements are related to accessing (mental) healthcare if you experience mental health issues. Mental health issues are issues that affect your mood, thoughts and/or behaviour. These can include, for instance, stress, worries, anxiety, or grief.  To what extent do you agree with the statements below? Please indicate the answer that best suits your personal opinion or experience. | | | | | |
| --- | --- | --- | --- | --- | --- |
|  | Strongly disagree | Disagree | Neither agree nor disagree | Agree | Strongly agree |
| **Availability of care** |  |  |  |  |  |
| I have access to resources (e.g. general practitioner/family caregiver, internet, friends) that I can use to gain access to receive care for mental health issues. | 1 | 2 | 3 | 4 | 5 |
| I have access to care for mental health issues in my mother tongue. | 1 | 2 | 3 | 4 | 5 |
| Professional help for mental health issues is not available in the area where I live. | 1 | 2 | 3 | 4 | 5 |
| Professional help for mental health issues is not available at the time required (e.g. inconvenient hours). | 1 | 2 | 3 | 4 | 5 |
| Professional help for mental health issues is not available in my preferred language. | 1 | 2 | 3 | 4 | 5 |
| **Approachability of care** |  |  |  |  |  |
| I know where to seek information about mental health issues. | 1 | 2 | 3 | 4 | 5 |
| I know how to use the computer to seek information about mental health issues. | 1 | 2 | 3 | 4 | 5 |
| I know how to use the telephone to seek information about mental health issues. | 1 | 2 | 3 | 4 | 5 |
| I am confident attending face-to-face appointments to seek information about mental health issues (e.g. seeing the general practitioner/family caregiver). | 1 | 2 | 3 | 4 | 5 |
| I know where to go to receive mental health services. | 1 | 2 | 3 | 4 | 5 |
| I experience practical problems (e.g. with things like transportation, childcare and/or scheduling) that prevent me from accessing (mental) healthcare services. | 1 | 2 | 3 | 4 | 5 |
| I know how or where to get help when I experience mental health issues. | 1 | 2 | 3 | 4 | 5 |
| **Acceptability of care** |  |  |  |  |  |
| I prefer to manage my mental health myself | 1 | 2 | 3 | 4 | 5 |
| I am afraid to ask for help when I experience mental health issues. | 1 | 2 | 3 | 4 | 5 |
| I am afraid of what others would think when they learn I experience mental health issues. | 1 | 2 | 3 | 4 | 5 |
| I do not bother to seek help when I experience mental health issues. | 1 | 2 | 3 | 4 | 5 |
| **Appropriateness of care** |  |  |  |  |  |
| Healthcare providers (e.g. doctors, nurses, therapists) know my wishes in terms of receiving mental healthcare. | 1 | 2 | 3 | 4 | 5 |
| Healthcare providers (e.g. doctors, nurses, therapists) know my needs in terms of receiving mental healthcare. | 1 | 2 | 3 | 4 | 5 |
| Providers respect the wishes that I have with regard to receiving mental healthcare. | 1 | 2 | 3 | 4 | 5 |
| Providers respect the needs that I have with regard to receiving mental healthcare. | 1 | 2 | 3 | 4 | 5 |
| Providers allow me to take part in my own care process. | 1 | 2 | 3 | 4 | 5 |
| Providers respect my cultural values. | 1 | 2 | 3 | 4 | 5 |

## Other study parameters (only for TCN participants)

| Below are some general questions and statements on your (mental) health, (mental) healthcare, and online information about (mental) health(care). To what extent do you agree with the statements below? Please indicate the answer that best suits your personal opinion or experience. | | | | | |
| --- | --- | --- | --- | --- | --- |
| **Current mental health status** | | | | | |
| How much of the time, during the last month, have you … | None of the time | A little of the time | Some of the time | Most of the time | All of the time |
| … been a very nervous person? | 1 | 2 | 3 | 4 | 5 |
| … felt calm and peaceful? | 1 | 2 | 3 | 4 | 5 |
| … felt downhearted and blue? | 1 | 2 | 3 | 4 | 5 |
| … been a happy person? | 1 | 2 | 3 | 4 | 5 |
| … felt so down in the dumps that nothing could cheer you up? | 1 | 2 | 3 | 4 | 5 |
| **Health literacy** |  | | | | |
| How often do you have someone help you read health materials? | Never | Occasionally | Sometimes | Often | Always |
| How confident are you filling out medical forms by yourself? | Not at all confident | A little bit confident | Somewhat confident | Quite a bit confident | Extremely confident |
| How often do you have problems learning about your medical condition because of difficulty in understanding written information? | Never | Occasionally | Sometimes | Often | Always |
| **eHealth literacy** |  | | | | |
|  | Strongly disagree | Disagree | Neither agree nor disagree | Agree | Strongly agree |
| I know *how* to find helpful health resources on the Internet. | 1 | 2 | 3 | 4 | 5 |
| I know *where* to find helpful health resources on the Internet. | 1 | 2 | 3 | 4 | 5 |
| I know how to use the Internet to answer my health questions. | 1 | 2 | 3 | 4 | 5 |
| I know what health resources are available on the Internet. | 1 | 2 | 3 | 4 | 5 |
| I know how to use the health information I find on the Internet to help me. | 1 | 2 | 3 | 4 | 5 |
| I have the skills I need to evaluate the health resources I find on the Internet. | 1 | 2 | 3 | 4 | 5 |
| I can tell high quality from low quality health resources on the Internet. | 1 | 2 | 3 | 4 | 5 |
| I feel confident in using information from the Internet to make health decisions. | 1 | 2 | 3 | 4 | 5 |

## Background characteristics

| **Background characteristics for TCN participants** | | | | | |
| --- | --- | --- | --- | --- | --- |
| How old are you? | __________ years old | | | | |
| What is your gender? | Male  Female  Other, namely: __________  I prefer not to say | | | | |
| What is your education level? | Primary or no education  Secondary (e.g. secondary vocational school, pre-university education)  Post-secondary (e.g. college, university of applied sciences, university, PhD)  Other, namely __________ | | | | |
| What is your country of birth? | __________ | | | | |
| What is your mother tongue?  *(feel free to list more than one, if applicable)* | __________ | | | | |
| What was your main reason for leaving your native country? | To flee from conflict, persecution or human rights violations  To flee from natural disaster (e.g. flood, hurricane or earthquake)  To find labour/work  To study  To reunite with my family  Other, namely: __________  I prefer not to say | | | | |
| In what country do you currently live? | Belgium  Germany  Italy  Lithuania  the Netherlands  Poland  Slovakia  Spain  the UK | | | | |
| How long have you been in this country? | __________ years | | | | |
| How fluent are you in the main language of the country you currently live, in terms of… | Not at all Completely fluent fluent | | | | |
| … speaking? | 1 | 2 | 3 | 4 | 5 |
| … listening? | 1 | 2 | 3 | 4 | 5 |
| … reading? | 1 | 2 | 3 | 4 | 5 |
| … writing? | 1 | 2 | 3 | 4 | 5 |

| **Background characteristics for HSCP participants** | |
| --- | --- |
| How old are you? | __________ years old |
| What is your gender? | Male  Female  Other / I do not wish to disclose |
| What is your country of birth? | __________ |
| What is your mother tongue?  *(feel free to list more than one, if applicable)* | __________ |
| In what country do you currently live? | Belgium  Germany  Italy  Lithuania  the Netherlands  Poland  Slovakia  Spain  the UK |
| What is your profession? | General practitioner/family doctor  Counsellor/therapist  Psychologist  Psychotherapist  Psychiatrist  Nurse  Social worker  Patient registration clerk/receptionist  Other, namely: __________ |
| How long have you worked in this profession? | < 1 year  1-5 years  6-10 years  11-15 years  > 15 years |
| How long have you worked with refugees or other migrants in this profession? | < 1 year  1-5 years  6-10 years  11-15 years  > 15 years |
| During the last 6 months, on average, how often did you work with refugees or other migrants? | At least once a day  At least once a week  At least once a month  Less than once a month |
| During the last 6 months, on average, how often did you experience communication difficulties due to language barriers during your job? | At least once a day  At least once a week  At least once a month  Less than once a month |
| Have you received any (postgraduate) training on intercultural competencies/communication? | No  Yes, namely __________ |

## End of the questionnaire

| Do you have any additional/final remarks? |  |
| --- | --- |

## Final page baseline questionnaire

| Thank you for taking the time to fill out this first questionnaire for the MHealth4All project!  If you feel the need to talk to someone due to this questionnaire, we advise you to contact a relative or friend, or your general practitioner/family caregiver.  You will now be asked to use the MHealth4All digital platform. This is a website which includes more information about accessing mental healthcare.  **The platform is available from: [INSERT WEBSITE HERE]**  You can use the same log-in information that you received to open this questionnaire.  You can use the platform for as long and as often as you would like.  The platform includes a brief web-lecture which explains how you can use it in your daily practice (e.g. with clients). In addition to this web-lecture, we will also ask you to participate in a communication training. The local research team in your host country has (most likely) already contacted you about this.    If you have any questions or remarks about the study, please contact one of the researchers. Their contact details are below.  Kind regards, on behalf of the MHealth4All study team,  [To be determined contact details per country]  More information about the MHealth4All project can be found on: <https://www.mhealth4all.eu/>. |
| --- |

# Posttest questionnaire

## Start page

| Dear Sir/Madam,  Thank you for your interest in participating in the MHealth4All study project. This project is sponsored by the European Union and is conducted in nine European countries (namely: Belgium, Germany, Italy, Lithuania, the Netherlands, Poland, Slovakia, Spain, and the United Kingdom). The goal of the project is to evaluate the effects of a newly developed digital platform. In particular, we want to know if the platform can help improve access to mental healthcare for refugees and other migrants.  You have previously filled out a first questionnaire on your ideas and beliefs on mental healthcare in the country you are currently in. You have also been requested to use the digital platform and to participate in an additional training. With this second questionnaire, we would like to focus on *your* personal experiences with the digital platform and its possible effects. There are no ‘right’ or ‘wrong’ answers. You would help us a lot by filling out this questionnaire using your first instinct or impressions. The questionnaire will take around 10-15 minutes to complete. People will not be able to trace your answers back to you or someone you care for.  We realize that the questionnaire may possibly evoke some emotions for you. If you feel upset or distressed, you do not have to continue with the questionnaire, or you can finish it another time. If you have filled out the questionnaire and feel the need to talk to someone, we advise you to contact a relative or friend, or your general practitioner/family caregiver.  Participation is voluntary. You are free to withdraw from the study at any given time. This will not disadvantage you in any way.  At the end of the questionnaire, there is the possibility to note additional thoughts or comments. If you have any questions or remarks about the study, please contact one of the researchers. Their contact details are below.  We greatly appreciate your participation and efforts.  Kind regards, on behalf of the MHealth4All study team,  [To be determined contact details per country]  More information about the MHealth4All project can be found on: <https://www.mhealth4all.eu/>. |
| --- |

## Start of the questionnaire

| **Please confirm whether you have received and been able to use the MHealth4All digital platform**  Yes, I have received the link to the MHealth4All digital platform  Yes, I have been able to use the MHealth4All digital platform  Yes, I have participated in the additional training |
| --- |

## Process evaluation of the platform

| Below are a few statements related to **videos** on the MHealth4All **digital platform**. To what extent do you agree with the statements below? Please indicate the answer that best suits your personal opinion or experience with the **videos**. | | | | | |
| --- | --- | --- | --- | --- | --- |
|  | Strongly disagree | Disagree | Neither agree nor disagree | Agree | Strongly agree |
| **Ease of use** |  |  |  |  |  |
| My interaction with the videos (e.g. clicking on hyperlinks) is clear and understandable. | 1 | 2 | 3 | 4 | 5 |
| I find the videos easy to use. | 1 | 2 | 3 | 4 | 5 |
| It is easy for me to become skilful skilled? at using the videos. | 1 | 2 | 3 | 4 | 5 |
| **Usefulness of the content** |  |  |  |  |  |
| I find the videos useful in my daily life/daily practice with patients/clients who experience language barriers. | 1 | 2 | 3 | 4 | 5 |
| Learning to use the insights from the videos (e.g. to gain access to mental healthcare/to mitigate language barriers) is easy for me. | 1 | 2 | 3 | 4 | 5 |
| **Comprehensibility of the information** |  |  |  |  |  |
| The videos are understandable for me. | 1 | 2 | 3 | 4 | 5 |
| The language in the videos is easy to comprehend for me. | 1 | 2 | 3 | 4 | 5 |
| The readability of the subtitles of the videos is good. | 1 | 2 | 3 | 4 | 5 |
| **Attractiveness of the content** |  |  |  |  |  |
| The videos are enjoyable. | 1 | 2 | 3 | 4 | 5 |
| The videos are creative. | 1 | 2 | 3 | 4 | 5 |
| The videos are clear and structured. | 1 | 2 | 3 | 4 | 5 |
| The videos are developed well. | 1 | 2 | 3 | 4 | 5 |
| The videos look nice. | 1 | 2 | 3 | 4 | 5 |
| **Emotional support** |  |  |  |  |  |
| The videos help me to deal with stress. | 1 | 2 | 3 | 4 | 5 |
| The videos help me with regulating my emotions. | 1 | 2 | 3 | 4 | 5 |
| The videos give me confidence. | 1 | 2 | 3 | 4 | 5 |
| The videos give me peace of mind. | 1 | 2 | 3 | 4 | 5 |

| Below are a few statements related to the **information portal** on the MHealth4All **digital platform**. To what extent do you agree with the statements below? Please indicate the answer that best suits your personal opinion or experience with the **information portal**. | | | | | |
| --- | --- | --- | --- | --- | --- |
|  | Strongly disagree | Disagree | Neither agree nor disagree | Agree | Strongly agree |
| **Ease of use** |  |  |  |  |  |
| My interaction with the information portal is clear and understandable. | 1 | 2 | 3 | 4 | 5 |
| I find the information portal easy to use. | 1 | 2 | 3 | 4 | 5 |
| It is easy for me to become skilful at using the information portal. | 1 | 2 | 3 | 4 | 5 |
| **Usefulness of the content** |  |  |  |  |  |
| I find the information portal useful in my daily life/daily practice with patients/clients who experience language barriers. | 1 | 2 | 3 | 4 | 5 |
| Learning to use the insights from the information portal (e.g. to gain access to mental healthcare/to mitigate language barriers) is easy for me. | 1 | 2 | 3 | 4 | 5 |
| **Comprehensibility of the information** |  |  |  |  |  |
| The information portal is understandable for me. | 1 | 2 | 3 | 4 | 5 |
| The language of the information portal is easy to comprehend for me. | 1 | 2 | 3 | 4 | 5 |
| The readability of the information portal platform is good. | 1 | 2 | 3 | 4 | 5 |
| **Attractiveness of the content** |  |  |  |  |  |
| The information portal is enjoyable. | 1 | 2 | 3 | 4 | 5 |
| The information portal is creative. | 1 | 2 | 3 | 4 | 5 |
| The information portal is clear and structured. | 1 | 2 | 3 | 4 | 5 |
| The information portal is developed well. | 1 | 2 | 3 | 4 | 5 |
| The information portal looks nice. | 1 | 2 | 3 | 4 | 5 |

| **Open-ended questions on the platform** |  |
| --- | --- |
| What (aspects of) the MHealth4All digital platform did you like, and why? For instance, what did you find (most) helpful or relevant? | ________________________ ________________________ ________________________ |
| What (aspects of) the MHealth4All digital platform could be improved or removed, and why? For instance, what did you find unhelpful or irrelevant? | ________________________ ________________________ ________________________ |
| What could we include in the MHealth4All digital platform to make it more helpful or relevant for you? For instance, is relevant information still missing? | ________________________ ________________________ ________________________ |
| Do you have any other thoughts, comments or suggestions for the MHealth4All digital platform? | ________________________ ________________________ ________________________ |

## Access to mental healthcare

| Below are a few statements related to accessing (mental) healthcare for refugees/migrants in the country where you live. All of these statements are related to accessing (mental) healthcare if you experience mental health issues. Mental health issues are issues that affect your mood, thoughts and/or behaviour. These can include, for instance, stress, worries, anxiety, or grief.  After using the digital platform, to what extent do you agree with the statements below? Please indicate the answer that best suits your personal opinion or experience. | | | | | |
| --- | --- | --- | --- | --- | --- |
|  | Strongly disagree | Disagree | Neither agree nor disagree | Agree | Strongly agree |
| **Availability of care** |  |  |  |  |  |
| I have access to resources (e.g. general practitioner/family caregiver, internet, friends) that I can use to gain access to receive care for mental health issues. | 1 | 2 | 3 | 4 | 5 |
| I have access to care for mental health issues in my mother tongue. | 1 | 2 | 3 | 4 | 5 |
| Professional help for mental health issues is not available in the area where I live. | 1 | 2 | 3 | 4 | 5 |
| Professional help for mental health issues is not available at the time required (e.g. inconvenient hours). | 1 | 2 | 3 | 4 | 5 |
| Professional help for mental health issues is not available in my preferred language. | 1 | 2 | 3 | 4 | 5 |
| **Approachability of care** |  |  |  |  |  |
| I know where to seek information about mental health issues. | 1 | 2 | 3 | 4 | 5 |
| I know how to use the computer to seek information about mental health issues. | 1 | 2 | 3 | 4 | 5 |
| I know how to use the telephone to seek information about mental health issues. | 1 | 2 | 3 | 4 | 5 |
| I am confident attending face-to-face appointments to seek information about mental health issues (e.g. seeing the general practitioner/family caregiver). | 1 | 2 | 3 | 4 | 5 |
| I know where to go to receive mental health services. | 1 | 2 | 3 | 4 | 5 |
| I experience practical problems (e.g. with things like transportation, childcare and/or scheduling) that prevent me from accessing (mental) healthcare services. | 1 | 2 | 3 | 4 | 5 |
| I know how or where to get help when I experience mental health issues. | 1 | 2 | 3 | 4 | 5 |
| **Acceptability of care** |  |  |  |  |  |
| I prefer to manage my mental health myself | 1 | 2 | 3 | 4 | 5 |
| I am afraid to ask for help when I experience mental health issues. | 1 | 2 | 3 | 4 | 5 |
| I am afraid of what others would think when they learn I experience mental health issues. | 1 | 2 | 3 | 4 | 5 |
| I do not bother to seek help when I experience mental health issues. | 1 | 2 | 3 | 4 | 5 |
| **Appropriateness of care** |  |  |  |  |  |
| Healthcare providers (e.g. doctors, nurses, therapists) know my wishes in terms of receiving mental healthcare. | 1 | 2 | 3 | 4 | 5 |
| Healthcare providers (e.g. doctors, nurses, therapists) know my needs in terms of receiving mental healthcare. | 1 | 2 | 3 | 4 | 5 |
| Providers respect the wishes that I have with regard to receiving mental healthcare. | 1 | 2 | 3 | 4 | 5 |
| Providers respect the needs that I have with regard to receiving mental healthcare. | 1 | 2 | 3 | 4 | 5 |
| Providers allow me to take part in my own care process. | 1 | 2 | 3 | 4 | 5 |
| Providers respect my cultural values. | 1 | 2 | 3 | 4 | 5 |

## Other parameters

| **Technology acceptance** | | | | | | | |
| --- | --- | --- | --- | --- | --- | --- | --- |
| Below are a few statements related to using health websites/digital platforms in general. To what extent do you agree with the statements below? Please indicate the answer that best suits your personal opinion or experience. | | | | | | | |
|  | Strongly Neither agree Strongly disagree nor disagree agree | | | | | | |
| I find health websites useful in my daily life. | 1 | 2 | 3 | 4 | 5 | 6 | 7 |
| Learning to use health websites is easy for me. | 1 | 2 | 3 | 4 | 5 | 6 | 7 |
| My interaction with health websites is clear and understandable. | 1 | 2 | 3 | 4 | 5 | 6 | 7 |
| I find health websites easy to use. | 1 | 2 | 3 | 4 | 5 | 6 | 7 |
| It is easy for me to become skilful at using health websites. | 1 | 2 | 3 | 4 | 5 | 6 | 7 |
| I intend to continue using health websites in the future. | 1 | 2 | 3 | 4 | 5 | 6 | 7 |
| I always try to use health websites in my daily life. | 1 | 2 | 3 | 4 | 5 | 6 | 7 |
| I will continue to use health websites frequently. | 1 | 2 | 3 | 4 | 5 | 6 | 7 |

## Background characteristics

| **Background characteristics** | | | | | |
| --- | --- | --- | --- | --- | --- |
| In what language did you use the digital platform? | [*We will include a list of all languages in which the multilingual platform has been made available*] | | | | |
| How fluent are you in the language in which you used the digital platform, in terms of… | Not at all Completely fluent fluent | | | | |
| … listening? | 1 | 2 | 3 | 4 | 5 |
| … reading? | 1 | 2 | 3 | 4 | 5 |

## End of the questionnaire

| Do you have any additional/final remarks? | _________________ _________________ _________________ |
| --- | --- |
| We would like to ask a few more questions to some of the participants on their experiences with the digital platform in a short interview (e.g. by telephone or online). May we contact you about this? | Yes  No |
| Would you like to be informed about the results of the MHealth4All project? | Yes  No |
| If you have answered ‘yes’ to one or both of the two questions above, you can leave your e-mail address here: | _________________ |

## Final page

| Thank you for taking the time to fill out this final questionnaire for the MHealth4All project!  If you feel the need to talk to someone due to this questionnaire, we advise you to contact a relative or friend, or your general practitioner/family caregiver.  If you want to use the digital platform again, you can continue to do so using the log-in details provided to you.  If you have any questions or remarks about the study, please contact one of the researchers. Their contact details are below.  Kind regards, on behalf of the MHealth4All study team,  [To be determined contact details per country]  More information about the MHealth4All project can be found on: <https://www.mhealth4all.eu/>. |
| --- |
