# Supplementary material for: Evaluating an intervention to promote access to mental healthcare for low language proficient migrants and refugees across Europe (MentalHealth4All): study protocol for a pretest–post-test cross-national survey study
Source: BMJ Open. 2025 Jul 7;15(7):e095868. doi: 10.1136/bmjopen-2024-095868 (PMC12258366; doi:10.1136/bmjopen-2024-095868)
Supplement: Supplementary file 2 [file bmjopen-2024-095868.reviewer_comments.pdf]

PEER REVIEW HISTORY

BMJ Open publishes all reviews undertaken for accepted manuscripts. Reviewers are asked to complete a checklist review form and are provided with free text boxes to elaborate on their assessment. These free text comments are reproduced below.

ARTICLE DETAILS

Title (Provisional)

Evaluating an Intervention to Promote Access to Mental Healthcare for Low Language Proficient Migrants and Refugees across Europe (MentalHealth4All): Study Protocol for a Pretest-Posttest Cross-National Survey Study

Authors

van Lent, Liza G.G.; Hodakova, Sona; Hanft-Robert, Saskia; Mösko, Mike; Rao, Chiara; Kerremans, Koen; Cox, Antoon; Lázaro Gutiérrez, Raquel; Temizöz, Özlem; Mankauskienė, Dalia; Biel, Łucja; Di Maria, Emilio; Schouten, Barbara; MentalHealth4All, Consortium

---

VERSION 1 - REVIEW

**Reviewer**	**1**
**Name**	**Sawadogo, Pengdewendé Maurice**
**Affiliation**	**Université Joseph Ki-Zerbo, Institut Supérieur des Sciences de la Population**
**Date**	**01-Feb-2025**
**COI**	**None**

P6 Line 25: How the nine European countries have been chosen?

P6 Line 40 : RQ2: How do participants (i.e. migrants/refugees and HSCPs) use the digital platform? Please clarify this research question to make it more precise.

P7 39-40 : How many years (months) will be considered

P11: Line 10-14: "personalised training session (either individual or in a group, and online or in-person) in which more detailed discussions will be held with a trainer on how to implement the (content of the) platform in the HSCPs' daily practice." The way to conduct this personalised training may be uniform.

P14: Statistical Analysis. For each dimension, you will use some items of a five point Likert Scale. How the value of each dimension computed using the items value? The score will be weighted or not?

P14: Precise the significance level for the test you will perform

P13 : LIne 51-52 : Justify why you "we strive to include around five times as many LLP migrants and refugees ".

VERSION 1 - AUTHOR RESPONSE

Reviewer: 1

Dr. Pengdewendé Maurice Sawadogo, Université Joseph Ki-Zerbo
Comments to the Author:
P6 Line 25: How the nine European countries have been chosen?
*Prior to the start of the project, deliberate efforts were made during the formation of the MentalHealth4All consortium to ensure broad geopolitical representation across Europe, including countries from the north, east, south, and west. The countries included in this study correspond with those represented in the MentalHealth4All consortium, with each participating country/university responsible for conducting the study in their own country. We have now included a brief clarification in the manuscript: "**These countries were selected as consortium partners to ensure broad geopolitical representation across northern, eastern, southern, and western Europe.**" (page 6-7 of the revised manuscript).*

P6 Line 40 : RQ2: How do participants (i.e. migrants/refugees and HSCPs) use the digital platform? Please clarify this research question to make it more precise.
*We have made the RQ more specific: "How do participants (i.e. migrants/refugees and HSCPs) use the digital platform **in terms of the pages/videos they access and average duration of the engagement**?" (page 6 of the revised manuscript)*

P7 39-40 : How many years (months) will be considered
*We are unsure to which lines the reviewer refers, but we have guessed that this question applies to one of our inclusion criteria for the providers/HSCPs. We have therefore specified that: "experience in delivering (mental) healthcare to migrants and refugees (i.e. at least one consultation with a LLP refugee/migrant client per month **on average in the past six months**);" (page 7 of the revised manuscript). If this is not correct, please let us know so that we can make further edits.*

P11: Line 10-14: "personalised training session (either individual or in a group, and online or in-person) in which more detailed discussions will be held with a trainer on how to implement the (content of the) platform in the HSCPs' daily practice." The way to conduct this personalised training may be uniform.
We thank the reviewer for pointing out this unclarity. Indeed, we developed a standardized outline for the personalized training session to make it more uniform across different trainers/countries. We have now specified this in the methods:
*"For the HSCPs, we have developed an accompanying**, standardised** training to teach them how to work with the platform, and how to integrate it into their clinical practice. The training consists of two parts:*

1. ***A** general online webinar for HSCPs on how they can use the platform in practice **– which is uploaded on the platform and available in the dominant languages of the nine participating countries;** and*
2. *a personalised**, structured** training session (either individual or in a group, and online or in-person) in which more detailed discussions will be held with a trainer on how to implement the*

(content of the) platform in the HSCPs' daily practice. ***A standard training outline and a model presentation were developed to promote consistency across trainers/countries.*** *"*

(page 9 of the revised manuscript)

P14: Statistical Analysis. For each dimension, you will use some items of a five point Likert Scale. How the value of each dimension computed using the items value? The score will be weighted or not?

As all dimensions will be analyzed separately, it is not necessary to weigh the different scores. For each separate dimension, we will compute the value by taking the average of the item scores (not weighted).

P14: Precise the significance level for the test you will perform

*We have now included a statement that "****We will use a significance level of p≤0.05 for all analyses.****" (page 14 of the revised manuscript).*

P13 : LIne 51-52 : Justify why you "we strive to include around five times as many LLP migrants and refugees ".

Initially – as part of the funding application – we conducted a power analysis for the providers (as described in the manuscript) and decided to aim for around five times as many migrants in order to link migrant experiences directly to their (trained) individual providers. However, after careful discussion with migrants (representatives) and providers, this linkage/approach proved to be infeasible for practical and logistical reasons. We still decided to maintain the original sampling ratio to ensure a more diverse representation of migrant experiences, which is essential for understanding variation in outcomes and needs across different backgrounds/characteristics. In other words, including about 260 migrants will – most likely – allow us to e.g. conduct various sensitivity analyses and to adjust for relevant covariates. We have now included a brief statement on this in the methods: "Additionally, we strive to include around five times as many LLP migrants and refugees (N=260) ***to ensure more diverse representation of migrants across different European countries, and to ensure sufficient power for e.g. sensitivity analyses and including relevant covariates****." (page 14 of the revised manuscript)*
